# Gut Peptide Alterations in Type 2 Diabetes and Obesity: A Narrative Review

**DOI:** 10.1007/s13679-026-00687-7

**Published:** 2026-01-23

**Authors:** Evangelia Tzeravini, Stamatia Simati, Ioanna A. Anastasiou, Maria Dalamaga, Alexander Kokkinos

**Affiliations:** 1 Diabetes Center, First Department of Propaedeutic Internal Medicine, Medical School, National and Kapodistrian University of Athens, Laiko General Hospital, 17 Agiou Thoma Street, Athens, 11527 Greece; 2 Department of Pharmacology, National and Kapodistrian University of Athens, Athens, Greece; 3 Department of Biological Chemistry, School of Medicine, National and Kapodistrian University of Athens, Athens, 11527 Greece

**Keywords:** Type 2 diabetes mellitus, Obesity, Proglucagon derived peptides, Gut derived peptides, Fasting, Postprandial

## Abstract

**Purpose of Review:**

The gastrointestinal tract acts as an endocrine organ, releasing hormones that regulate glucose homeostasis, appetite, energy expenditure, and gastrointestinal motility. In type 2 diabetes (T2DM) and obesity, this finely tuned hormonal system is disrupted, contributing to metabolic dysfunction. This review summarizes current evidence on fasting and postprandial responses to mixed-meal tests (MMT) and oral glucose tolerance tests (OGTT) of proglucagon-derived peptides (PGDPs), orexigenic and anorexigenic hormones, and less frequently studied gastrointestinal peptides in individuals with T2DM and obesity compared with healthy controls.

**Recent Findings:**

Studies demonstrate that while GLP-1 levels are often preserved, its insulinotropic and glucagonostatic actions are impaired in T2DM and obesity. GIP secretion is maintained or increased but exhibits reduced biological efficacy. Oxyntomodulin and GLP-2 show blunted postprandial responses, whereas glicentin, GRPP, and MPGF remain poorly characterized but appear dysregulated. PYY is reduced in obesity and shows impaired postprandial rises in T2DM, while PP is frequently elevated in T2DM. CCK resistance may diminish satiety signaling, though secretion data are inconsistent. Secretin and amylin exhibit complex, stage-specific alterations, whereas ghrelin and obestatin are typically reduced in both conditions.

**Summary:**

Gut hormone alterations in T2DM and obesity include both adaptive and pathogenic features, reflecting disruptions across multiple peptide systems. Standardization of peptide measurements and deeper investigation into their mechanistic roles will be essential for advancing precision-based interventions targeting gastrointestinal hormones in metabolic disease.

## Introduction

The gastrointestinal tract is traditionally recognized for its roles in nutrient digestion and absorption; however, it also functions as a complex endocrine organ. Specialized enteroendocrine cells distributed along the gut secrete a wide range of peptides, collectively known as gut hormones or gut peptides, which orchestrate critical processes in glucose metabolism, appetite regulation, gastrointestinal motility, and energy homeostasis [1]. These peptides have attracted increasing attention due to their relevance in the pathophysiology and treatment of obesity, type 2 diabetes mellitus (T2DM), and related metabolic disorders.

Despite intense focus on incretins such as (glucagon-like peptide-1) GLP-1 and glucose-dependent insulinotropic polypeptide (GIP), comprehensive evaluation of other gut peptides—including proglucagon-derived peptides (PGDPs) beyond GLP-1 and GIP, as well as orexigenic and anorexigenic hormones—remains lacking.

Among the most studied gut-derived peptides are the incretins: GLP-1 and GIP. GLP-1, produced by intestinal L-cells in response to food intake, enhances insulin secretion, suppresses glucagon, delays gastric emptying, and promotes satiety [2]. GIP, secreted by K-cells, similarly stimulates insulin release but loses efficacy in advanced T2DM [3]. Therapeutics based on these peptides—including GLP-1 receptor agonists and dual GLP-1/GIP receptor agonists like tirzepatide—have shown significant benefits in glycemic control and weight reduction [4].

GLP-1, along with other PGDPs such as oxyntomodulin (OXM), glucagon-like peptide-2 (GLP-2), glicentin, and glucagon, is encoded by the proglucagon (*Gcg*) gene [5]. In pancreatic alpha cells, proglucagon is processed by prohormone convertase 2 (PC2) to generate glucagon, glicentin-related pancreatic polypeptide (GRPP), intervening peptide-1 (IP-1), and major proglucagon fragment (MPGF) [5]. In contrast, in intestinal L-cells and specific neurons, prohormone convertase 1/3 (PC1/3) mediate the production of GLP-1, GLP-2, OXM, glicentin, and IP-2 [5]. Interestingly, alpha cells may also express PC1/3, contributing to local islet-derived GLP-1 that augments insulin secretion in a paracrine manner [6]. Each PGDP has distinct roles: GLP-2 supports intestinal health, glucagon regulates blood glucose and appetite, OXM acts on both GLP-1 and glucagon receptors to reduce appetite and body weight, and glicentin influences gut growth and motility [4, 6].

Beyond PGDPs, other gut-derived peptides such as peptide YY (PYY), pancreatic polypeptide (PP), secretin (SCT), amylin, ghrelin, and obestatin contribute to metabolic regulation. PYY and PP are key satiety signals, while SCT is involved in appetite suppression, bile secretion, and thermogenesis [7–9]. Amylin, co-secreted with insulin, modulates postprandial glucose levels and gastric emptying but may exacerbate beta cell stress when dysregulated [10]. Ghrelin, a stomach-derived hormone, stimulates appetite and opposes insulin action, whereas obestatin, derived from the same precursor as ghrelin, may counteract its orexigenic effects [11]. Notably, ghrelin and PYY influence not only appetite but also insulin secretion and action, with ghrelin inhibiting and PYY potentially supporting beta cell function over time [12, 13].

There is growing evidence that the secretion and action of these peptides are altered in metabolic disease. T2DM is characterized by hyperglucagonemia, impaired postprandial suppression of glucagon, and reduced responsiveness to incretins—a phenomenon known as the “incretin defect” [14]. Although the secretion of GLP-1 and GIP may not be markedly reduced, beta cell sensitivity to their insulinotropic effects is diminished [14]. In contrast, exogenous GLP-1 at pharmacological doses can overcome this resistance, unlike GIP [15]. Patients with T2DM also display blunted postprandial responses of OXM, glicentin, and PYY, along with paradoxically lower fasting ghrelin levels, possibly reflecting hormonal resistance or compensatory adaptations [16–18]. Moreover, fasting ghrelin has been inversely correlated with insulin sensitivity, further linking it to metabolic dysregulation [19].

Despite their biological importance, many gut-derived peptides remain understudied in clinical contexts, and their secretion dynamics, receptor distributions, and physiological actions differ substantially between healthy individuals and those with obesity or T2DM, with important therapeutic implications. Although alterations in individual gut peptides have been widely reviewed, no prior review has comprehensively integrated evidence on all gut-derived peptides across both obesity and T2DM, and analyses of fasting and postprandial dynamics across peptide classes remain limited. In this narrative review, we synthesize current evidence on classical incretins and less frequently examined gastrointestinal hormones—including PGDP beyond GLP-1 and GIP, as well as orexigenic and anorexigenic peptides—comparing individuals with obesity or T2DM with healthy controls. Importantly, we examine gut peptide responses not only in the fasting state but also following physiological stimuli, distinguishing findings from mixed-meal tests (MMT) and oral glucose tolerance tests (OGTT) and highlighting the influence of meal composition on postprandial hormone secretion. By integrating dispersed literature across experimental conditions and peptide classes, this review aims to clarify inconsistencies in reported responses and to provide a physiological framework relevant to emerging multi-agonist incretin-based therapies.

## Literature Search

This is a narrative review, and the literature search was conducted up to June 2025 to capture relevant evidence on gut peptide alterations in obesity and T2DM rather than to perform a systematic review. A search was performed using PubMed and Google Scholar, as well as the reference lists of relevant review and research articles. Key search terms included “obesity,” “type 2 diabetes mellitus,” “GLP-1,” “GLP-2,” “proglucagon,” “glucagon,” “oxyntomodulin,” “glicentin,” “major proglucagon fragment or MPGF,” “glicentin-related pancreatic polypeptide or GRPP,” “GIP,” “incretins,” “peptide YY,” “cholecystokinin,” “secretin,” “amylin,” “ghrelin,” “obestatin,” “fasting,” “postprandial,” and “meal.” Only original research articles and meta-analyses published in English were included. No strict time frame was applied; however, very old studies (pre-1980) or studies that used measurement methods no longer considered valid were generally excluded when more recent, reliable evidence was available, with a few exceptions when data were limited. This search strategy was used to capture fasting and postprandial data on both classical and less-studied gut peptides in individuals with obesity or T2DM. Given the breadth of the available literature, it was not possible to discuss all relevant studies in detail. Therefore, emphasis was placed on representative, recent and methodologically robust evidence.

## The PGDPs

### Glucagon

Glucagon is a 29-amino acid peptide hormone produced by pancreatic alpha cells through the action of PC2 on proglucagon, with trace amounts also detected in brain neurons [5]. Its primary stimulus is hypoglycemia, though mixed meals, amino acids, and various peptides and hormones also promote its release [5]. Notably, sympathetic and parasympathetic signaling, along with neuropeptides, enhance glucagon secretion [5]. Glucagon exerts autocrine effects on alpha cells to stimulate its own production, while insulin, somatostatin, gamma-aminobutyric acid (GABA), amylin, serotonin, and GLP-1 suppress its release [5, 20]. It has a short half-life, with hepatic and renal clearance playing major roles in its metabolism [5]. Upon secretion, glucagon binds to its G-protein-coupled receptor (GCGR), primarily expressed in the liver and kidneys, leading to increased cyclic AMP (cAMP) levels [5]. This cascade promotes glycogenolysis and gluconeogenesis while inhibiting glycolysis and glycogenesis, resulting in elevated blood glucose levels [5]. Glucagon also stimulates fatty acid oxidation and ketogenesis under low-glucose conditions [5]. However, its role in glucose metabolism is complex, as GCGRs are also found in beta cells, where glucagon enhances glucose-mediated insulin secretion [5]. Recent data suggest that glucagon may support glucose production during fasting but promote insulin secretion postprandially [6]. On the other hand, newer data support that the insulin-secreting action of glucagon is mediated by its binding to the GLP-1R receptor on beta cells, while the increase in glucose results from the stimulation of GCGR in extra-pancreatic tissues [6]. Additionally, miniglucagon, a glucagon-derived peptide, inhibits insulin release, potentially modulating metabolic responses [21]. Beyond glucose regulation, glucagon suppresses appetite, increases energy expenditure, and has cardioprotective effects, possibly through brown adipose tissue activation and lipolysis [5].

There is substantial evidence supporting that patients with T2DΜ or impaired glucose tolerance (IGT) exhibit mildly elevated fasting glucagon levels [22–28]. Under conditions of poor glycemic control or diabetic ketoacidosis, glucagon levels are significantly higher [29–32]. Moreover, individuals with T2DΜ or IGT appear to have a reduced suppression of glucagon secretion by glucose [22, 24, 27, 28, 33–36], or mixed meals [36–39] compared to healthy controls. Consequently, both fasting and postprandial glucagon levels have been negatively associated with insulin sensitivity [35, 40, 41]. Faerch et al. found that in T2DΜ, insufficient early glucagon suppression followed by delayed intense suppression (30–120 min post-glucose) correlates with insulin resistance [22]. Others have reported similar early suppression deficits [25], while Wagner et al. found that reduced glucagon at 120 min correlated with higher insulin sensitivity in non-diabetic individuals, revealing inconsistencies in the data [42]. On the other hand, glucagon response to arginine or glucose has been linked to future risk of developing IGT according to two prospective studies [43, 44]. However, other studies have found no differences in fasting glucagon levels [35, 38, 45], or in glucagon response following glucose ingestion [46] or a MMT [47] between individuals with T2DΜ and healthy controls. Additionally, most studies on postprandial glucagon response in T2DΜ have focused on values obtained after OGTT or carbohydrate-rich meals [22, 25, 33, 37, 48]. This highlights a gap in the literature regarding glucagon fluctuations after fat- and protein-rich meals in individuals with T2DΜ and prediabetes. Given that amino acids and fatty acids stimulate glucagon secretion while carbohydrates suppress it [49], further research is needed to explore alpha cell responses to meals of varying macronutrient composition.

Additionally, in individuals with diabetes, glucagon secretion in response to hypoglycemia is diminished, indicating alpha cell dysfunction [50]. Thus, T2DΜ is associated with elevated fasting glucagon levels and abnormal glucagon responses to both hyperglycemic and hypoglycemic states.

Hyperglucagonemia is observed in obesity, with studies reporting higher fasting [47, 51–55] and post-MMT or post-OGTT glucagon levels correlated with BMI [47, 54–56]. Stefanakis et al. noticed that fat-rich meals induced a greater incremental area under the curve (iAUC) and slower glucagon decline than carbohydrate-rich meals [52]. Similarly, Knop et al. found that although fasting glucagon levels were higher in individuals with obesity and normal glucose tolerance, glucagon responses to OGTT did not differ from those of lean subjects [14]. Several studies have also reported no abnormalities in fasting glucagon [23] or postprandial glucagon areas under the curve (AUC) [34] in individuals with obesity versus their lean counterparts. There is currently no consensus regarding the postprandial glucagon response in obesity. It has also been suggested that obesity and hepatic fat deposition may be more strongly associated with hyperglucagonemia than T2DM [51].

### OXM and Glicentin

OXM is a 37-amino acid peptide containing glucagon and IP-1, secreted mainly by intestinal L-cells postprandially, with additional central nervous system (CNS) expression [57]. It is rapidly degraded by dipeptidyl peptidase-4 (DPP-4) [57]. Although no specific receptor has been identified, OXM binds weakly to GLP-1 and glucagon receptors, thereby modulating glucose metabolism [57, 58]. It may stimulate glucose production and enhance intestinal glucose absorption via the glucagon receptor, while promoting insulin secretion and weight loss through GLP-1 receptor activation [57–59]. Exogenous OXM improves glycemic control and insulin secretion in both animals and humans with obesity or T2DM, although its concurrent glucagon receptor activity may attenuate its glucose-lowering effects relative to GLP-1 receptor agonists [57, 59]. OXM also stimulates endogenous glucagon, partially offsetting its insulinotropic effect [57].

Beyond glycemia, OXM reduces food intake and increases energy expenditure, contributing to weight loss that may surpass GLP-1 receptor agonists [57, 59, 60]. These effects appear to be mediated by both the glucagon and GLP-1 receptors [57, 59, 61]. It also suppresses appetite and reduces gastric and pancreatic secretions [57].

Glicentin, comprising GRPP, glucagon, and IP-1, is also secreted by L-cells in response to nutrients [62]. It supports intestinal growth, slows motility, and suppresses gastric acid secretion [62]. Glicentin may enhance insulin secretion and inhibit glucagon, though its mechanism remains unclear, with possible action via GLP-1 receptors [62, 63]. Fasting glicentin levels have been associated with activation of brain reward centers, suggesting a role in appetite regulation [64].

Limited data are currently available regarding the impact of diabetes and prediabetes on endogenous OXM levels. In a study by Wewer Albrechtsen et al., both OXM and glicentin levels in the blood following an OGTT were significantly lower in the T2DM group compared to the control group [17]. However, in the same study, no differences were observed between individuals with and without obesity [17]. Similarly, Stafeev et al. found that fasting and postprandial OXM levels were lower in patients with T2DM and obesity compared to those with obesity alone, without diabetes [65]. It is worth noting, however, that in this study, patients with diabetes underwent a MMT, whereas the control group underwent an OGTT [65]. Furthermore, in patients with newly diagnosed prediabetes or diabetes due to acute pancreatitis, postprandial OXM levels after an MMT were approximately 24% lower than in healthy controls [66]. On the other hand, Bharmal et al. observed that OXM levels after a MMT were lower in patients with diabetes or prediabetes secondary to pancreatitis compared to those with pre-existing T2DM or prediabetes before the onset of pancreatitis [67]. They suggested that OXM could be used in the differential diagnosis of these conditions [67]. In both diabetic and prediabetic groups, postprandial OXM levels were lower than in healthy controls [67].

To our knowledge, studies that compared directly oxyntomodulin levels between subjects with obesity and those with normal weight are meager. Two research groups however failed to show any significant difference in OXM levels between lean subjects and those with obesity [17, 68].

With regard to glicentin, various findings indicate that patients with IGT or T2DM have lower fasting glicentin levels than healthy controls [69]. Notably, in one study, even acute IGT due to acute pancreatitis was associated with reduced fasting glicentin levels [70]. Manell et al. observed significantly lower postprandial glicentin levels after an OGTT in adolescents with obesity and IGT, and marginally lower levels when obesity was combined with T2DM, compared to individuals with obesity alone [69]. As previously mentioned, post-OGTT glicentin levels in adults with T2DM were also lower than in healthy controls [17]. In the study by Manell et al., fasting glicentin levels had good predictive value for diagnosing IGT in adolescents with obesity and normal fasting glucose level [69]. Similarly, Hoffmann et al. monitored patients with prediabetes over a year and observed that although there were no initial differences, those who developed T2DM during the study exhibited lower post-OGTT glicentin and GLP-1 stimulation, reduced postprandial glucagon suppression, and longitudinal changes in glicentin and GLP-1 AUCs that were predictive of T2DM onset [16]. However, other researchers failed to demonstrate a statistically significant association between fasting glicentin and insulin resistance [71].

Individuals with obesity also appear to have lower fasting [71] and postprandial [52, 68] glicentin levels compared to lean controls, although this observation is not consistently confirmed across studies [17, 69].

In summary, both OXM and glicentin tend to be lower in individuals with diabetes and/or obesity. However, it should be noted that the measurement of both OXM and glicentin have been technically challenging, requiring years of research to develop reliable methodologies. As a result, comparisons between studies utilizing different measurement techniques remain difficult.

### GLP-1

GLP-1 is primarily secreted by intestinal L-cells in response to food intake, with a biphasic release pattern postprandially [2]. Factors such as meal composition and size influence GLP-1 secretion, with lipids inducing a delayed but prolonged response [2]. Additionally, GLP-1 is secreted at basal levels during fasting and may also be regulated by the autonomic nervous system and ghrelin [2]. Beyond the intestine, GLP-1 production has been identified in pancreatic alpha cells under metabolic stress and in certain neurons of the central nervous system [2]. However, its biological activity is rapidly terminated by degradation via DPP-4, with only 10–15% of the secreted hormone reaching the pancreas [2].

GLP-1 exerts its effects through the GLP-1 receptor (GLP-1R), which is primarily expressed in pancreatic beta and delta cells, as well as in neurons, myocardial cells, and other tissues [72]. Its primary function is to enhance glucose-dependent insulin secretion while suppressing glucagon release, thereby reducing hepatic glucose production and maintaining glycemic homeostasis [2, 73]. It also protects beta cells by inhibiting apoptosis, slows gastric emptying, reduces appetite via central mechanisms, and may influence lipid metabolism [2, 72, 73]. Emerging evidence also suggests roles in cardiovascular and renal function [74]. However, while GLP-1-based therapies are effective for glycemic control and weight loss, their full physiological impact requires further elucidation [73].

Several studies have reported a reduced basal [75] and postprandial secretion of GLP-1 in patients with T2DM, however, recent meta-analyses have not confirmed a significant impairment in GLP-1 secretion in individuals with T2DM or prediabetes compared to healthy controls, whether assessed after OGTT or a mixed meal [76–78]. Calanna et al. observed in a post-hoc analysis that higher glycated hemoglobin (A1C) levels were associated with a lower iAUC for GLP-1 [76]. Conversely, in some studies, patients with T2DM who had a shorter disease duration and lower A1C levels exhibited a higher postprandial GLP-1 response compared to healthy controls, possibly as a compensatory mechanism due to reduced GLP-1 action [76, 79]. This suggests that glycemic control and diabetes duration may influence postprandial GLP-1 secretion [76].

The meta-analysis by Watkins et al. indicated that differences in GLP-1 measurement methods might account for discrepancies between studies comparing individuals with T2DM and controls [78]. Additionally, medications such as metformin and colesevelam, which increase GLP-1 levels, may contribute to the heterogeneity observed across studies [80–82]. Furthermore, although not extensively studied, GLP-1 secretion appears to vary by sex and age, potentially affecting study outcomes. For instance, Færch et al. found a positive correlation between lower GLP-1 levels in individuals with prediabetes or T2DM and female sex, attributing this partially to higher GLP-1 levels in female controls compared to males [83]. The same study reported higher GLP-1 levels in older individuals, possibly due to impaired renal function and reduced GLP-1 clearance [83].

Even if GLP-1 levels remain unaffected by diabetes status, evidence suggests that its insulinotropic effect and suppression of glucagon secretion are impaired in individuals with T2DM compared to both healthy controls and those with IGT [34, 84] However, some studies have reported no significant difference in GLP-1-mediated glucagon suppression between T2DM patients and healthy individuals [85]. Other researchers have noted a general incretin defect in T2DM and prediabetes, without differentiating whether this is due to impaired GLP-1 function, GIP function, or both [86].

In individuals with obesity, postprandial GLP-1 secretion appears to be reduced following OGTT [87], independent of the presence of IGT or overt T2DM [34, 83]. GLP-1 levels after a MMT have been negatively associated with body weight in some studies [87–90], while others found no correlation [91, 92]. Interestingly, Perakakis et al. and Bowen et al. observed a positive correlation between BMI and both fasting and postprandial GLP-1 levels [54, 93]. It is also possible that basal active GLP-1 secretion is diminished in obesity [94]. Weight loss through dietary interventions improves postprandial GLP-1 levels, though they do not reach those observed in individuals with normal body weight [89, 95]. However, some studies, such as those by Sumithran et al., have reported no significant changes in GLP-1 levels despite one year of weight loss [96]. Sloth et al. observed an initial decrease in GLP-1 levels after eight weeks of dieting, but levels returned to baseline by six months [97].

A study on monozygotic and dizygotic twins suggested that approximately 67% of the GLP-1 response after OGTT is genetically determined [98]. In the same study, GLP-1 levels after both OGTT and MMT were lower in twins with higher body weight compared to their leaner siblings, with associations to insulin resistance and hepatic fat deposition [98]. The authors concluded that obesity alone is insufficient to impair incretin responses; other metabolic syndrome components may also contribute [98]. However, a limitation of this study was the lack of glucagon measurements. Muscelli et al. found that incretin function was negatively correlated with both body weight and the presence of T2DM, independently of one another [34]. Some researchers, however, have found no significant differences in GLP-1 levels between individuals with obesity and those with normal weight [99]. Collectively, the available data suggest that postprandial GLP-1 secretion may be impaired in obesity; however, whether this is a consequence of increased body weight or contributes to the pathogenesis of obesity remains unclear.

### GLP-2

Glucagon-like peptide-2 (GLP-2), a 33-amino acid peptide co-secreted with GLP-1, which exhibits a biphasic release pattern and is rapidly inactivated by DPP-4 [100]. Though not a major regulator of glucose, GLP-2 maintains intestinal integrity via its receptor (GLP-2R), promoting enterocyte proliferation, reducing apoptosis, and enhancing barrier function and blood flow [100]. It also decreases gastric acid and intestinal motility, though its physiological relevance remains under investigation [100].

GLP-2 also affects the pancreas, with pharmacological doses stimulating glucagon secretion in humans and mice, though results are inconsistent under varying glycemic conditions [100, 101]. In GLP-2R-deficient ob/ob mice, elevated glucagon levels, alpha cell mass, and hyperglycemia were observed, along with reduced beta cell mass, indicating a complex role in obesity and glucose regulation [101].

Additional GLP-2 effects include reduced bone resorption, enhanced lipid absorption, and CNS-mediated regulation of blood pressure and neuroprotection [100]. Although rodent data suggest anorexigenic effects, peripheral GLP-2 analogs do not significantly alter food intake in humans [100, 102].

Few studies have compared GLP-2 levels between individuals with diabetes and healthy controls. In a study by Cazzo et al., the GLP-2 AUC following a MMT was significantly lower in participants with both T2DM and class I obesity, as well as in those with class III obesity without diabetes, compared to lean, healthy controls [103]. Conversely, Gelonese et al. reported a negative correlation between postprandial GLP-2 levels and insulin sensitivity [104]. However, in a study by Higgins et al., while fasting GLP-2 levels were not associated with insulin resistance, postprandial GLP-2 responses were negatively correlated with homeostatic model assessment of insulin resistance (HOMA-IR) [105].

Obesity appears to be associated with a reduced postprandial GLP-2 response [103], possibly due to increased DPP-4 activity [87], which may partly explain the findings of Cazzo et al. In contrast, fasting GLP-2 levels were significantly higher in individuals with obesity compared to lean individuals in two other studies [52, 106], while a third study found no difference in fasting GLP-2 levels between individuals with elevated versus normal body weight [105]. The macronutrient composition of test meals used in different studies may also influence outcomes, as protein-rich meals appear to elicit a greater GLP-2 increase compared to carbohydrate-rich meals and, even more so, compared to high-fat meals [107].

### MPGF and GRPP

Few researchers have measured MPGF levels across different populations, and data on GRPP are nearly nonexistent. Since MPGF contains the GLP-1 sequence in its structure, older analytical methods may have falsely measured it as GLP-1 [45]. Stefanakis et al. examined individuals with obesity, overweight, and normal body weight and observed a positive correlation between MPGF—both fasting and postprandial—and body mass index (BMI) [54]. Weight loss following a three-month treatment with either liraglutide or bupropion/naltrexone resulted in a reduction of the postprandial MPGF response, with the effect being more pronounced in the bupropion/naltrexone group and independent of changes in body weight. However, this reduction correlated negatively with changes in lean mass [108]. Notably, the decrease in fasting glucose levels following the three-month intervention and weight loss was negatively associated with the reduction in MPGF AUC [108].

Similarly, in a recent study by the same group, no differences in MPGF levels were observed between lean individuals and those with obesity when consuming a balanced, low-calorie meal [52]. However, following a high-calorie, high-fat meal, the postprandial MPGF response was greater in the obesity group [52]. Additionally, participants in the high-fat meal study arm exhibited differences in fasting MPGF levels, with higher values observed in the obesity group [52]. MPGF levels also showed a positive correlation with body weight, body fat and male sex [52]. In the same study, a comparison between a high-fat meal and a carbohydrate-rich meal revealed that MPGF exhibited a more prolonged elevation following fat consumption [52].

Furthermore, in a study by Polyzos et al., where participants underwent liver biopsy, MPGF levels were positively associated with hepatic steatosis in the early stages of metabolic dysfunction-associated fatty liver disease (MAFLD) [109]. On the other hand, Kokkinos et al. did not observe a significant difference in either fasting or postprandial MPGF levels between patients with obesity and lean ones [68]. Direct evidence comparing MPGF levels between individuals with and without diabetes is lacking, and the same is true for GRPP.

## Other Gut Peptides

### GIP

The GIP gene, located on chromosome 17, encodes proGIP, which is processed to the active form GIP (1–42) by PC 1/3 [3]. GIP is secreted by the K-cells of the small intestine in response to nutrients, particularly fats and carbohydrates, and has a short half-life due to DPP-4 degradation [3]. Unlike GLP-1, GIP remains more stable and is also cleared by the kidneys [110].

GIP promotes insulin secretion, synthesis, beta cell proliferation, and survival [3]. It also stimulates glucagon release, contributing to glucose regulation under both hypoglycemic and hyperglycemic conditions [3]. Thus, GIP plays a complementary role in maintaining glucose homeostasis [3]. Unlike GLP-1, GIP does not slow gastric emptying but can inhibit gastric acid secretion at high concentrations [111]. GIP receptors are expressed in the CNS, bone, and adipose tissue, where GIP promotes anabolic effects and may enhance bone formation [3]. Its influence on memory, liver fat, and appetite regulation remains less clearly defined [3, 112].

In individuals with T2DM, both basal and postprandial GIP secretion has been reported to be slightly increased in some studies [34, 113, 114]. However, a meta-analysis of 22 studies indicated no significant difference in GIP secretion following an OGTT or MMT between individuals with and without T2DM, a finding also reported for GLP-1 [115]. According to the same meta-analysis, BMI was positively associated with the GIP response, whereas age and A1C levels showed a negative correlation [115]. Given the heterogeneity among studies and measurement methodologies for GIP, it remains possible that as T2DM progresses, GIP synthesis may ultimately decline. Even if GIP production is maintained, all available evidence suggests that in individuals with T2DM of various etiologies, GIP loses its ability to stimulate insulin secretion from beta cells [15, 116, 117]. This loss of GIP’s incretin effect could be either a consequence or a cause of diabetes. Evidence suggests that the impairment in GIP-induced insulin secretion occurs after, rather than before, the onset of insulin resistance, making it more likely a consequence of the metabolic disorder leading to T2DM [118, 119]. Two potential mechanisms have been proposed to explain the loss of GIP function: a reduction in beta cell mass due to the progression of diabetes and glucotoxicity or a decrease in GIP receptor (GIPR) expression on beta cells [120]. On the other hand, improving glycemic control to near-normal levels may partially, but not fully, restore the GIP’s incretin action [121, 122].

GIP has an anabolic effect on adipose tissue, promoting subcutaneous fat deposition while also enhancing the release of pro-inflammatory cytokines from adipose tissue—disturbances commonly observed in obesity [3]. However, data on the effect of obesity on GIP levels remain limited. Some evidence suggests that individuals with overweight/obesity exhibit increased fasting and postprandial GIP secretion following an OGTT or MMT [14, 99, 123–125]. A positive correlation between body weight and GIP levels has also been observed in patients with T2DM [115]. Conversely, other studies have reported no difference [87, 89, 98, 126–128] in GIP levels in individuals with obesity compared to lean ones. The discrepancies among studies may be attributed to differences in methodology and GIP measurement techniques.

### PYY and PP

PYY, composed of 36 amino acids, is primarily produced by intestinal L-cells and plays a key role in appetite regulation and metabolism [7]. It is released in response to food intake, reaching peak levels two hours postprandially, with its secretion influenced by meal composition and vagal nerve activity [7]. PYY(1–36) is metabolized by DPP-4 into PYY(3–36), its more active form, which primarily binds to Y2 receptors in the brain, suppressing appetite by inhibiting agouti-related peptide and neuropeptide Y (AgRP/NPY) neurons and activating proopiomelanocortin (POMC) neurons [7]. Additionally, PYY increases energy expenditure by enhancing thermogenesis, reduces gastric motility, and increases intestinal absorption of water and sodium [7, 129].

Regarding its effects on metabolism, PYY appears to suppress glucose-stimulated but not basal insulin secretion and also inhibits lipolysis [13]. The exact mechanism by which PYY reduces glucose-induced insulin secretion remains unclear, though both direct and indirect actions on beta cells have been proposed [13]. Experimental studies suggest that PYY administration enhances insulin sensitivity [13, 130]. While both forms exhibit anorexigenic effects, PYY(1–36) plays a greater role in insulin suppression, whereas PYY(3–36) primarily enhances insulin sensitivity [13]. Together, they ultimately lower insulin levels and insulin resistance, although this effect is not yet well-documented [13]. Overall, it contributes to energy balance and has been proposed as a potential therapeutic target for obesity and diabetes [13].

The available data so far are inconclusive regarding the actual status of PYY levels in individuals with T2DM. Fasting PYY levels have been found to be elevated in individuals with T2DM or IGT compared to healthy controls [131, 132], and they correlated positively with A1C levels [131]. In another study however, first degree relatives of patients with T2DM had lower basal PYY than the control group, which was negatively correlated with insulin resistance [133]. English et al. observed that while fasting PYY was higher in individuals with T2DM, the postprandial PYY response was attenuated compared to weight-matched healthy controls [132]. In individuals with a combination of obesity and T2DM/IGT, the PYY response to fat intake was also diminished compared to the control group; however, in this case, the coexistence of severe obesity may have influenced the results [134]. Conversely, the PYY AUC following a ΜΜΤ was higher in individuals with T2DM compared to those with IGT; however, this study did not include a comparison with healthy controls [135]. Additionally, Viardot et al. reported a lower PYY response after a carbohydrate-rich meal in individuals with a family history of T2DM but without IGT, compared to those without a family history of T2DM, whereas neither fasting PYY levels nor PYY AUC after a fat-rich meal differed between the two groups [136]. Furthermore, according to Belinova et al., meal composition plays a role in the PYY response [137]. A carbohydrate-based meal induced greater PYY responses in individuals with T2DM compared to a fat- and protein-rich meal, while the opposite was observed in healthy controls [137].

However, other studies have not found a significant correlation between fasting PYY and insulin resistance [138, 139], while Brownley et al. reported a positive correlation between PYY AUC and insulin sensitivity [140].

On the other hand, existing data consistently indicate that PYY levels, both fasting [141–145], and postprandial [140–144, 146, 147] are lower in individuals with obesity compared to lean individuals. PYY has also been found to be negatively associated with BMI [139, 141]. There does not appear to be resistance to PYY action in individuals with obesity, as exogenous administration led to appetite suppression similar to that observed in healthy controls [142]. Additionally, the ratio of PYY(1–36) to PYY(3–36) did not differ between individuals with normal and increased body weight, suggesting an overall reduction in PYY production in obesity rather than a selective decrease in its fractions [142]. Given that PYY suppresses appetite, its lower concentrations in individuals with obesity may contribute to increased food intake and weight gain. However, some researchers did not observe a significant difference in fasting PYY levels [140, 147–150] or postprandial PYY levels [145, 149] or following an OGTT [150] between individuals with and without obesity. Others paradoxically found a positive correlation between PYY levels and body weight [131]. Stock et al., despite not recording a significant difference in PYY AUC after a mixed meal between individuals with obesity and controls, observed an earlier postprandial increase in PYY at 15 min in healthy controls, which was absent in the obesity group [149].

Overall, based on the above, PYY has a strong anorexigenic effect, and its levels are likely suppressed in individuals with obesity, which may at least partially explain their increased food intake and weight gain. Conversely, exogenous PYY administration has been found to reduce appetite [141]. Finally, in individuals with T2DM, there is evidence of elevated fasting PYY levels but an inadequate postprandial increase. PYY suppresses insulin secretion while simultaneously enhancing tissue insulin sensitivity. Therefore, its diminished postprandial response may be linked to the hypersecretion of insulin observed in individuals with T2DM and insulin resistance.

Pancreatic polypeptide (PP) is a 36-amino acid peptide secreted by F-cells of the pancreatic islets in response to food intake, particularly fats, though other factors such as cholecystokinin (CCK), GIP, adrenaline, and somatostatin also regulate its release [9]. PP levels remain elevated for about six hours post-secretion and it is metabolized by DPP-4 and neprilysin [9]. It binds to Y4 receptors in the brainstem, hypothalamus, and other tissues, reducing appetite, pancreatic exocrine secretion, gastric emptying, and gallbladder motility [9]. In mice, PP has been linked to increased energy expenditure, likely due to enhanced locomotion [9]. While it suppresses insulin secretion, it also exerts a protective effect on beta cells [151]. PP administration in animals, and humans, including those with Prader-Willi syndrome, reduces appetite and food intake [151].

Limited human studies have shown that fasting and postprandial PP levels are elevated in individuals with T2DM compared to healthy controls. Among T2DM patients, those not receiving insulin therapy exhibit higher PP levels than insulin-treated individuals [152]. In diabetes secondary to chronic pancreatitis, both basal and postprandial PP secretion are increased compared to patients with chronic pancreatitis without diabetes [153]. However, in individuals who developed diabetes or prediabetes after acute pancreatitis, PP secretion does not differ from healthy controls [154]. Another study reported higher PP levels in T2DM patients without pancreatic disease compared to those with diabetes due to pancreatic exocrine dysfunction [155]. Weight loss in T2DM patients is associated with reduced PP levels, and PP changes correlate inversely with insulin sensitivity [156].

In Prader-Willi syndrome-related obesity, both fasting and postprandial PP levels are reduced [157]. However, findings in non-syndromic obesity are inconsistent, with some studies reporting decreased fasting and postprandial PP levels [158, 159], while others found no difference compared to lean individuals [160]. Diet-induced weight loss has been linked to increased fasting PP in children with obesity [158], and exercise has been shown to enhance postprandial PP response [161]. In contrast, a study by Kahleova et al. reported that diet-induced weight loss led to reduced fasting and postprandial PP levels in adults with T2DM, with no additional effect from exercise [156].

### CCK

CCK is produced by I cells in the duodenum, as well as by neurons in the gut and brain [8, 162]. Food intake, particularly lipids and proteins, stimulates CCK secretion, which subsequently binds to CCK-1 receptors in peripheral tissues and CCK-2 receptors in the CNS [8]. CCK receptors are G protein-coupled, and their activation facilitates nutrient absorption in the intestine, slows gastric emptying and gastric acid secretion, stimulates the exocrine pancreas to produce digestive enzymes, enhances gallbladder contraction, reduces energy intake, and increases insulin secretion [8, 162]. Many of these effects are mediated by activation of the vagus nerve, suppression of ghrelin, and stimulation of leptin by CCK [8, 162].

In individuals with obesity, there appears to be resistance of vagal neurons to CCK, leading to a diminished anorexigenic effect [145] and perhaps impaired CCK secretion. However, a recent meta-analysis found no significant differences in CCK levels between lean individuals and people with obesity [163]. On the other hand, limited data exist regarding potential CCK dysregulation in individuals with T2DM compared to healthy individuals. Milewicz et al. did not observe differences in fasting CCK levels between participants with and without T2DM, although CCK was positively correlated with leptin and insulin [164]. Notably, findings by Rhee et al., who analyzed intestinal biopsies, indicated that CCK mRNA expression and the density of CCK-producing cells were similar between samples from patients with T2DM and those from healthy controls [165]. In contrast, another study found that while fasting CCK levels did not differ between individuals with and without T2DM, the postprandial CCK response was significantly lower in the diabetes group [166]. However, an opposing set of findings was reported by another research team [167], highlighting the inconsistency of available data and the difficulty in drawing definitive conclusions.

### Secretin

Secretin (SCT) is a 27-amino acid peptide primarily produced by S-cells in the proximal small intestine, with additional expression in enteroendocrine and dendritic cells [168, 169]. Its secretion is stimulated by acidic gastric contents, lipids, and proteins, while the role of carbohydrates remains unclear [168, 169]. Prolonged fasting increases SCT levels, and a secretin-releasing peptide has been identified [169]. SCT has a short half-life (2.5–4 min) and is primarily cleared by the kidneys [169].

SCT exerts its effects through the secretin receptor (SCTR), a G protein-coupled receptor widely expressed in the body [168, 169]. Its primary function is to stimulate pancreatic exocrine secretion, while it also inhibits gastric acid secretion and gastric emptying [168, 169]. In the hepatobiliary system, SCT promotes bicarbonate-rich bile secretion and relaxation of the sphincter of Oddi, facilitating bile flow into the duodenum [168]. Emerging evidence suggests a role for SCT in appetite regulation, as both central and peripheral SCT administration suppresses food intake, possibly via activation of POMC neurons in the arcuate nucleus and the vagus nerve [170]. SCT also exhibits neuroprotective properties and may be critical for brain development [168, 169].

SCT has a weak incretin effect, primarily influencing early-phase glucose-mediated insulin secretion rather than insulin synthesis [171]. However, its insulinotropic action appears significant only at supraphysiological concentrations [171]. It may also suppress glucagon release under hyperglycemic conditions but not during euglycemia [172]. Additionally, SCT has been implicated in thermogenesis via UCP-1 activation in brown adipose tissue [173], and in lipolysis in white adipose tissue [174]. Beyond metabolic regulation, SCT has been associated with increased cardiac output, stroke volume, and coronary and renal blood flow while reducing peripheral vascular resistance [168, 169]. It may also play a role in airway hydration, mucus clearance, smooth muscle relaxation, and whole-body water balance [168, 169].

In patients with DM, SCT-induced insulin secretion has been found to be reduced compared to healthy controls [175–177], although some researchers have reported no significant difference between individuals with and without DM [178, 179]. A recent study by Gilliam-Vigh et al., using intestinal biopsies, also demonstrated that SCT synthesis, SCTR mRNA expression, and the density of S-cells did not differ between patients with T2DM and healthy volunteers [180]. On the other hand, secretin release in response to duodenal acidification was found lower in patients with DM compared with the control group [177]. On the contrary, Trimble et al., reported higher fasting as well as post OGTT secretin values in subjects with newly diagnosed T2DM compared with healthy controls, although glucose consumption during OGTT suppressed secretin secretion in both groups [181].

In individuals with obesity, there is evidence that the SCT increase in response to prolonged fasting, as well as insulin secretion following an OGTT in response to exogenous SCT administration, is lower compared to normal weight individuals [182]. However, other studies have found no significant differences in fasting SCT levels or postprandial SCT response following a high-fat meal across individuals with varying body weights [183, 184], except in cases where obesity coexisted with IGT, in which case the incretin effect of SCT was diminished compared to healthy controls [184]. Conversely, according to Erk et al., individuals with obesity required lower doses of exogenous SCT to elicit insulin release compared to normal weight individuals [179].

Most of the aforementioned studies on individuals with obesity and DM date back several decades, and SCT measurements were performed using radioimmunoassay (RIA), which detected porcine SCT. This technique has been criticized for its reliability, potentially explaining the inconsistencies in the reported findings [185]. Therefore, the development of more accurate methods for SCT measurement is necessary to enable reliable comparisons across different populations.

### Amylin

Amylin (formerly known as diabetes-associated peptide) is a 37-amino acid peptide encoded by the *IAPP* (islet amyloid polypeptide) gene [10]. It is co-secreted with insulin by pancreatic beta cells, as well as by enteroendocrine and neural cells [10]. Within pancreatic islets, amylin is stored with insulin and released in response to food intake, regulated by signals such as GLP-1 [10, 186]. Structurally similar to calcitonin, amylin acts via its receptor, formed by the heterodimerization of the calcitonin receptor with receptor activity-modifying proteins (RAMPs 1–3) [10]. Initially synthesized as a prohormone, it is activated through proteolytic processing by proconvertases, akin to PGDPs [186].

Amylin suppresses appetite by acting directly on the brain and enhancing leptin’s effects, while also promoting increased energy expenditure [10, 187]. It plays a dual role in glucose homeostasis and T2DM progression. It suppresses postprandial glucagon release, hepatic glucose production, pancreatic enzyme secretion, and gastric emptying, lowering postprandial blood glucose levels [186]. However, in early T2DM, excessive amylin production due to insulin resistance leads to amyloid aggregation, contributing to beta cell dysfunction and disease progression [10]. Beyond its metabolic functions, amylin may have neuroprotective and cardiovascular effects, while also being implicated in amyloid-related diseases, such as Alzheimer’s [186, 187].

With regard to T2DM, Harter et al., observed that individuals with T2DM on insulin treatment exhibited lower fasting amylin levels, as expected considering that exogenous insulin suppresses b-cell insulin/amylin co-excretion, while subjects on oral antidiabetic medications had higher basal amylin values than the control group [188]. In the same study, amylin response to OGTT was higher in the group with diabetes [188]. On the other hand, Hanabusa et al., reported similar fasting amylin in T2DM- lean patients on oral hypoglycemic agents and lower fasting amylin in insulin-treated T2DM subjects, along with decreased amylin responses to OGTT compared with healthy controls [189]. Fasting amylin values were also found to be lower in T2DM patients on metformin and higher in those on glibenclamide, in comparison to healthy controls [190]. Higher fasting [191–193], as well as lower post OGTT [193, 194] and higher post MMT [79] amylin levels have been reported by other research groups. Some studies however did not observe differences in either fasting [194, 195] or postprandial [195] amylin secretion in subjects with T2DM compared with healthy controls. It is important to highlight that all the aforementioned studies involved patients receiving treatment with metformin, sulfonylureas, insulin, or dietary interventions. The impact of newer antidiabetic agents on amylin secretion remains unexplored. Additionally, during the early stages of T2DM, insulin resistance and compensatory hyperinsulinemia may lead to elevated amylin secretion. As beta cell dysfunction progresses, however, the production of both insulin and amylin declines. This dynamic may, at least in part, account for the inconsistencies observed across existing studies.

Trials in humans have demonstrated that individuals with obesity exhibit elevated fasting amylin levels [191, 194–196] and greater postprandial amylin responses compared to lean individuals [194, 196, 197], even when obesity co-exists with T2DM on oral hypoglycemic agents [189]. A positive correlation has also been reported between amylin levels and both body weight [198] and insulin resistance [196]. This suggests that amylin may either increase in parallel with body weight as a compensatory mechanism aimed at reducing adiposity or that obesity-induced resistance to amylin’s effects leads to its upregulation. Notably, weight loss through diet and exercise has been shown to reduce fasting amylin levels in both normal-weight individuals and people with obesity [199], and similar were the results with combined training in a group of patients with T2DM [198].

### Ghrelin and Obestatin

Ghrelin was initially identified for its role in growth hormone (GH) regulation rather than appetite stimulation [200]. While GH release is primarily regulated by growth hormone-releasing hormone (GHRH) and somatostatin, ghrelin acts independently through the GH secretagogue receptor (GHS-R) [11]. It is mainly produced in the stomach but is also synthesized in the pancreas, placenta, and other tissues [11]. Ghrelin is derived from preproghrelin and becomes active through acylation by the enzyme ghrelin O-acyltransferase (GOAT) [201]. It circulates in both acylated (active) and desacylated (inactive) forms, with secretion influenced by fasting, insulin, and body weight [11].

Ghrelin binds to GHS-R1a in the hypothalamus and peripheral tissues, stimulating GH secretion and appetite via AgRP/NPY neurons [11]. It also activates AMP-activated protein kinase (AMPK) and interacts with vagal and dopaminergic pathways [11]. In addition to promoting food intake, ghrelin reduces energy expenditure by suppressing thermogenesis in brown adipose tissue and increasing fat storage [11].

In the pancreas, ghrelin may inhibit glucose-stimulated insulin secretion from beta cells, resulting in higher blood glucose levels [12]. Exogenous ghrelin administration in humans has shown similar effects [202]. The mechanism is unclear but may involve somatostatin signaling and reduced intracellular calcium in beta cells [203]. Ghrelin may also contribute to insulin resistance through increased free fatty acid release and hepatic gluconeogenesis [204].

Though not essential for growth, ghrelin plays a key role in maintaining glucose homeostasis and preventing hypoglycemia during energy deficiency [205]. Beyond metabolism, ghrelin supports cardiovascular health, enhances gastric motility, and shows neuroprotective effects in models of Parkinson’s disease [206].

Desacyl ghrelin, previously thought inactive, may counteract acyl ghrelin by improving insulin sensitivity and modulating fat distribution [207].

While ghrelin promotes weight gain in rodents [208], paradoxically, individuals with obesity tend to have lower fasting ghrelin levels than lean individuals [209, 210]. Lean individuals have higher fasting ghrelin levels than those with obesity [211, 212], with the highest levels observed in patients with anorexia [213, 214]. This paradox has led to the hypothesis of ghrelin resistance, characterized by decreased ghrelin receptor (GHS-R) expression in the hypothalamus and reduced AgRP/NPY peptide production [215]. Additionally, while total and acylated ghrelin levels are lower in obesity, the enzyme GOAT—responsible for ghrelin acylation—appears to be upregulated in severe obesity, possibly as a compensatory response to low ghrelin levels [216]. On the other hand, postprandial suppression of ghrelin, a crucial mechanism for satiety, is impaired in obesity, contributing to prolonged food intake and increased caloric consumption [147, 217].

Obesity is often associated with insulin resistance and hyperinsulinemia, which may explain the inverse relationship between ghrelin and BMI. Several studies have reported lower fasting ghrelin levels in patients with T2DM compared to healthy controls [137, 218, 219] and many data support a negative correlation between fasting ghrelin and insulin resistance, independently of BMI [19, 220, 221]. Postprandial ghrelin responses in T2DM remain understudied. Some findings suggest that individuals with T2DM exhibit diminished postprandial ghrelin suppression and a transient postprandial drop, potentially linked to fasting ghrelin levels [137, 222]. On the contrary, Erdmann et al., reported that diabetes alone did not influence postprandial ghrelin responses [219]. Notably, lower fasting ghrelin levels correlate with an increased risk of T2DM and hypertension, even in individuals with a family history of T2DM [223]. Genetic factors also play a role, with specific polymorphisms in the ghrelin and GHS-R genes linked to increased susceptibility to obesity and T2DM [223–227].

Most evidence indicates that weight loss increases ghrelin levels in individuals with obesity [228, 229], while others found neutral effect of weight loss on ghrelin [230, 231]. However, once body weight stabilizes post-weight loss, ghrelin tends to return to its original low levels [232].

Obestatin is a 23-amino acid peptide derived from the proghrelin molecule, similar to ghrelin, with the stomach as its primary site of synthesis [233, 234]. It is also secreted in smaller amounts by the intestine and pancreatic ε-cells [233, 234]. Obestatin binds to the orphan receptor GPR39, expressed in various tissues, including the gastrointestinal tract, liver, adipose tissue, pituitary gland, and hypothalamus [233, 234]. However, its low specificity for GPR39 makes it unlikely to mediate its effects through this receptor. Instead, obestatin exhibits high affinity for GLP-1R, as well as for HIT-T15 and INS-1E beta cell receptors, though its precise mechanism of action remains unclear [233, 234].

Initial studies suggested that obestatin suppressed appetite and food intake in mice [235], leading to its classification as a ghrelin antagonist. However, subsequent research failed to confirm its anorexigenic effects [236, 237], and showed no impact on pituitary hormones such as GΗ [238], or leptin [235]. Lagaud et al. reported a U-shaped relationship between obestatin levels and appetite, with no anorexigenic effects observed at either high or low doses, potentially explaining conflicting study results [239]. Animal and ex vivo experiments further suggest a dose-dependent interaction between obestatin and insulin [233, 234].

Obestatin administration appears to have a dose-dependent effect on insulin secretion, inhibiting glucose-stimulated insulin release at low doses while enhancing it at higher doses [240]. Additionally, glucose levels influence obestatin’s impact on beta cells [233]. Evidence also suggests that obestatin slows gastric emptying, promotes beta cell survival, increases pancreatic juice secretion, stimulates glucagon release, inhibits somatostatin secretion, and may play a role in sleep regulation, memory function, and thirst suppression [233, 234].

Limited data are available on obestatin levels in individuals with metabolic syndrome. Reduced fasting obestatin [241] and postprandial [242] concentrations have been reported in patients with T2DM and insulin resistance while a positive correlation between fasting obestatin and insulin sensitivity has been observed in healthy adults [243, 244]. Fasting obestatin levels are also lower in individuals with obesity compared to those with normal body weight [245–247]. Postprandial obestatin responses were also lower in people with obesity than in lean individuals [245]. One study reported an increased ghrelin-to-obestatin ratio in individuals with obesity [248], whereas another has observed the opposite trend [249], leading to inconclusive findings.

**Fig. 1:**
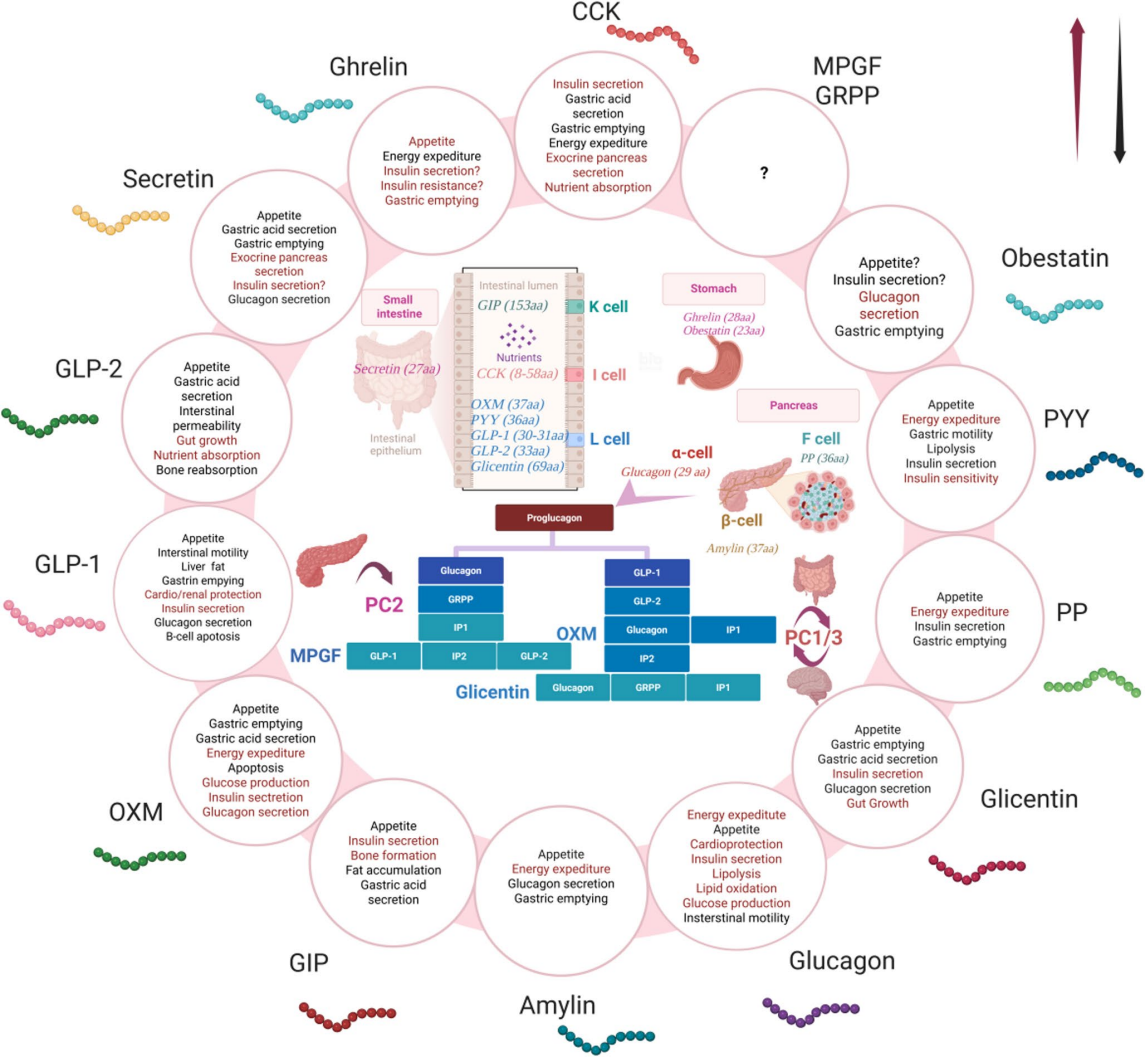
Secretion sites and action of key gut-derived peptides. Abbreviations: OXM: oxyntomodulin, GLP-1: Glucagon-like peptide-1, GLP-2: Glucagon-like peptide-2, CCK: cholecystokynin, MPGF: major proglucagon fragment, GRPP: glicentin-related pancreatic polypeptide, PYY: peptide tyrosin-tyrosin, PP: pancreatic polypeptide, GIP: Glucose-dependent insulinotropic polypeptide, PC: prohormone convertase, IP-1: intervening peptide-1, aa: amino acids. Graphical symbols used in the figure: ↑ and dark red indicate stimulatory actions of gut peptides, whereas ↓ and black indicate inhibitory actions;? denotes insufficient or inconclusive evidence

The main findings of this review are summarized in Fig. 1; Table 1.

**Table 1:** Fasting and postprandial values of gut derived peptides in patients with T2DM and obesity

Peptide	T2DM		Obesity	
	Fasting	Post-prandial	Fasting	Post-prandial
OXM	↓[65]	↓[65] MMT for T2D & OGTT for controls ↓[17] OGTT	↔[68]	↔[17] OGTT ↔[68] MMT
Glicentin	↓[45, 69] ↔[71]	↓[16, 17] OGTT	↓[71] ↔ [68, 69]	↔ [17, 69] OGTT ↓[108] MMT
GLP-2	No data	↓[103] MMT	↑[52, 106] ↔[105]	↓[103] MMT ↔[105] high fat meal
MPGF	No data	No data	↑[54] ↔[68]	↑[54] MMT ↑high-cal/fat meal [52] ↔low-cal/fat meal [52] ↔[68] MMT
GLP-1	↓[75]	↔[76–78] MMT & OGTT meta-analyses	↑[54, 93]	↓[87–90] MMT ↓[34, 83, 98] OGTT ↔[92, 98] MMT ↓high-carbo/↔high fat meal [91] ↑[54, 93] MMT
GIP	↑[34] ↔[113, 114]	↔[115]MMT & OGTT meta-analyses	↔[14, 87, 89, 98, 123–128] ↑[99]	↔[87, 89, 126, 127] MMT ↔[98, 127, 128] OGTT ↑[99, 123–125] MMT ↑[14] OGTT
Glucagon	↑[22–29] ↔[38, 45]	↑[22, 24, 27, 28, 34, 36] OGTT ↑[36–39] MMT ↔[46] OGTT ↔[47] MMT	↑[14, 47, 51–55] ↔[23]	↑[47, 54–56] MMT ↔[14, 34] OGTT
PYY	↑[131, 132] ↔[134] T2D with obesity	↓[132, 134] MMT or high fat meal ↓[136] in IGT after high carbo ↓[137] high fat meal ↑[137] high carbo meal	↓[141–145] ↔[139, 140, 146–150]	↓[140–144, 146, 147] MMT ↔[150] OGTT ↔[145, 149] MMT
PP	↑[153] chronic pancreatitis related DM	↑[153] MMT in chronic pancreatitis related DM ↔[154] MMT in acute pancreatitis related DM	↓[158, 159] ↔[160]	↓[159] MMT ↔[160] MMT
CCK	↔[164, 166, 167]	↓[166] MMT ↑[167] liquid meal	↔[163]	↔[145] MMT ↔[163] meta-analysis
Secretin	↔[180] SCT mRNA ↑[181]	↑[181] OGTT	↔[183, 184]	↔[183] liquid fatty meal
Amylin	↑[188] & ↔[189] on OHA, ↓[188, 189] on insulin ↑[191–193] OHA ↔[194, 195] ↑on metformin/↓on SU[190]	↑[188] OGTT ↑[79] MMT ↔[195] OGTT ↓[189, 194] OGTT	↑[189, 191, 194–196]	↑[79, 196] MMT ↑[189, 194, 195, 197] OGGT
Obestatin	↓[241] ↔[242]	↑[242] MMT	↓[245–247]	↓[245]MMT
Ghrelin	↓[137, 218, 219]	↓[137] but impaired suppression after MMT ↓[218] OGTT ↔[219] MMT	↓[209, 210, 212, 217] ↔[147]	↓[217, 219] but impaired suppression after MMT ↑[147] MMT

**Abbreviations**: *carbo* carbohydrates, *CCK* cholecystokynin, *GIP* Glucose-dependent insulinotropic polypeptide, *GLP-1* Glucagon-like peptide-1, *GLP-2* Glucagon-like peptide-2, *MMT* mixed-meal test, *MPGF* major proglucagon fragment, *OGTT* oral glucose tolerance test, *OHA* oral hypoglycemic agents, *OXM* oxyntomodulin, *PP* pancreatic polypeptide, *PYY* peptide tyrosin-tyrosin, *SU* sulfonylureas, *T2DM* Type 2 diabetes mellitus

↑, ↓ and ↔ denote increased, decreased or similar levels of the examined peptide between subjects with T2D or obesity and healthy controls

## Clinical Translation: Bariatric Surgery and Emerging Pharmacotherapies

Alterations in endogenous gut peptide secretion have direct clinical relevance, particularly in the context of bariatric surgery and emerging pharmacotherapies for obesity and T2DM. Each bariatric procedure is characterized by a distinct hormonal signature, with Roux-en-Y gastric bypass (RYGB) and sleeve gastrectomy (SG) inducing rapid and pronounced increases in postprandial gut hormone secretion [232, 250]. Enhanced GLP-1 and PYY responses after RYGB and SG act synergistically to promote satiety, reduce food intake, and improve glycemic control, with GLP-1 also contributing to restoration of the impaired incretin effect in T2DM [129, 232]. These hormonal adaptations occur early after surgery, preceding substantial weight loss, although their interpretation is partly confounded by concurrent caloric restriction and later by weight-loss–dependent effects.

Beyond classical incretins, postprandial changes in less-studied PGDPs such as oxyntomodulin and glicentin have emerged as potential markers of surgical outcomes. Postprandial glicentin and oxyntomodulin responses have been associated with postoperative weight loss after bariatric surgery [250, 251], while fasting glicentin levels may predict postprandial hypoglycemia following RYGB [252]. Similarly, post-meal GLP-1 and PYY dynamics have correlated with long-term weight loss outcomes after SG [253]. These findings suggest that specific endogenous gut peptide profiles may help identify individuals at risk of suboptimal weight loss or metabolic complications and could serve as early biomarkers to guide postoperative monitoring and supportive interventions.

Insights from these surgery-induced endocrine adaptations have directly informed the development of modern pharmacotherapies for obesity and T2DM. While bariatric procedures induce coordinated increases in multiple gut peptides, pharmacological strategies aim to reproduce key elements of this hormonal milieu without surgery [254].

Early efforts to target glucagon signaling achieved glucose lowering but were limited by adverse effects, including hypoglycemia and hepatic toxicity. In contrast, the amylin analogue pramlintide has achieved clinical use as an adjunct to insulin therapy, exerting beneficial metabolic effects through delayed gastric emptying and appetite suppression in addition to indirect glucagon inhibition [186]. GLP-1 receptor agonists now represent a cornerstone of therapy for both T2DM and obesity, with consistent benefits on glycemic control, body weight, and cardiovascular and renal outcomes [129, 255]. The transition from short-acting exendin-based compounds to long-acting human GLP-1 analogues has improved therapeutic durability, while newer dual incretin agonists, such as GLP-1/GIP receptor agonists, achieve superior metabolic efficacy by engaging complementary hormonal pathways [129, 255]. Building on the physiology of bariatric surgery and endogenous peptides such as oxyntomodulin, emerging dual and triple agonists targeting GLP-1, GIP, and glucagon receptors seek to combine appetite suppression, improved glycemic control, and increased energy expenditure. Early clinical data suggest potential benefits for obesity, T2DM, and metabolic-associated steatotic liver disease [255]. However, interindividual variability in treatment response remains incompletely understood. Importantly, while baseline glucagon levels may influence glycemic responses to certain oral glucose-lowering agents, such as acarbose [256], evidence directly linking baseline or postprandial endogenous gut peptide levels to responsiveness to exogenous incretin therapies is currently lacking. Pharmacological studies of GLP-1 receptor agonists and dual GLP-1/GIP agonists indicate that clinical characteristics—including baseline body weight, BMI, HbA1c, age, sex, and treatment duration—are currently stronger predictors of therapeutic response than endogenous peptide levels [257]. Taken together, these findings highlight the need for future studies to directly evaluate whether baseline or postprandial gut peptide profiles can inform individualized treatment strategies for obesity and T2DM, particularly in the context of multi-agonist therapies.

## Conclusions

The gastrointestinal tract serves as a crucial endocrine organ, releasing hormones that regulate glucose metabolism, appetite, energy balance, and gastrointestinal motility. In T2DM and obesity, this hormonal network is disrupted, leading to altered secretion and impaired action of key peptides. Importantly, PGDPs like oxyntomodulin, GLP-2, GLP-1, glicentin, MPGF, and GRPP are increasingly recognized as modulators of gut–pancreas signaling. Oxyntomodulin and GLP-2 responses appear blunted in metabolic disease, while data on glicentin, MPGF, and GRPP remain limited but suggest disrupted secretion. Importantly, most studies on these peptides have been conducted in European or North American populations, and data from non-European cohorts are scarce, highlighting a clear need for research in ethnically diverse populations to determine whether gut peptide dynamics differ across populations. GLP-1 levels are often preserved in T2DM, but its insulinotropic and glucagon-suppressing effects are diminished. In obesity, postprandial GLP-1 responses are inconsistently reduced.

GIP secretion is generally intact or slightly increased in both conditions, yet its metabolic efficacy is blunted, likely due to beta cell or receptor dysfunction. Anorexigenic peptides such as PYY and PP also show altered dynamics. Obesity is associated with reduced fasting and postprandial PYY levels, while T2DM shows blunted postprandial rises despite variable fasting levels. PP is often elevated in T2DM but less well-studied in obesity. Other hormones —including CCK, secretin, amylin, ghrelin, and obestatin— exhibit condition-specific changes. While CCK resistance may impair satiety in obesity and T2DM, inconsistent findings regarding its secretion and expression limit definitive conclusions. Secretin and amylin have complex roles in metabolism, with amylin initially elevated and later deficient in T2DM. Ghrelin and obestatin levels are reduced in both diseases, potentially reflecting resistance and impaired metabolic control.

These hormone alterations may represent both adaptive responses and drivers of metabolic dysfunction. By integrating fasting and postprandial evidence across classical and less-studied gut peptides and distinguishing responses to mixed meals and oral glucose challenges, this review provides a physiological framework for interpreting gut hormone dysregulation in obesity and T2DM and for contextualizing emerging multi-agonist incretin therapies.

## Future Perspectives

Despite growing evidence of altered gut peptide secretion in obesity and T2DM, important knowledge gaps remain. Future studies should expand investigation beyond classical incretins to include less-studied PGDPs (oxyntomodulin, glicentin, MPGF, GRPP, GLP-2) and other gastrointestinal hormones such as CCK, secretin, and obestatin, ideally across diverse populations and metabolic phenotypes.

Progress will require more standardized and physiologically relevant study designs. Harmonization of mixed-meal and OGTT protocols, together with methodological consistency in peptide measurement, including validated assays and concurrent assessment of multiple gut peptides within the same study, will be essential to improve comparability and to capture coordinated hormonal responses.

Most existing studies emphasize circulating hormone levels rather than functional relevance. Integrating fasting and postprandial gut peptide profiles with outcomes related to appetite, energy intake, and glucose metabolism is necessary to clarify their physiological and clinical significance. Finally, building on insights from bariatric surgery and incretin-based therapies, future research should directly examine whether endogenous gut peptide patterns can predict responsiveness to GLP-1–based, dual, or multi-agonist treatments, thereby supporting more personalized therapeutic approaches in obesity and T2DM.

## Annotated References

- Knop FK, Aaboe K, Vilsboll T, Volund A, Holst JJ, Krarup T et al. Impaired incretin effect and fasting hyperglucagonaemia characterizing type 2 diabetic subjects are early signs of dysmetabolism in obesity. Diabetes Obes Metab 2012; 14: 500–10.
  - ○ Demonstrates that impaired incretin action and dysregulated glucagon secretion are early abnormalities in obesity and T2DM, supporting the central role of gut peptides in pathogenesis.
- Watkins JD, Carter S, Atkinson G, Koumanov F, Betts JA, Holst JJ et al. Glucagon-like peptide-1 secretion in people with versus without type 2 diabetes: a systematic review and meta-analysis. Metabolism 2023; 140: 155375.
  - ○ Meta-analysis confirming that GLP-1 secretion is not consistently reduced in T2DM, but its functional activity is impaired, highlighting the importance of receptor responsiveness.
- Calanna S, Christensen M, Holst JJ, Laferrere B, Gluud LL, Vilsboll T et al. Secretion of glucose-dependent insulinotropic polypeptide in patients with type 2 diabetes: systematic review and meta-analysis. Diabetes Care 2013; 36: 3346–52.
  - ○ Provides comprehensive evidence that GIP secretion is preserved in T2DM, but its insulinotropic effect is blunted, reinforcing the concept of “GIP resistance.”
- Matikainen N, Bogl LH, Hakkarainen A, Lundbom J, Lundbom N, Kaprio J et al. GLP-1 responses are heritable and blunted in acquired obesity with high liver fat and insulin resistance. Diabetes Care 2014; 37: 242–51.
  - ○ Highlights genetic and metabolic influences on GLP-1 response, linking impaired secretion to obesity-associated fatty liver and insulin resistance.
- Wewer Albrechtsen NJ, Hornburg D, Albrechtsen R, Svendsen B, Torang S, Jepsen SL et al. Oxyntomodulin identified as a marker of type 2 diabetes and gastric bypass surgery. EBioMedicine 2016; 7: 112–20.
  - ○ Demonstrates reduced oxyntomodulin in T2DM and normalization post-bariatric surgery, suggesting its potential as a biomarker of metabolic improvement.
- Manell H, Staaf J, Manukyan L, Kristinsson H, Cen J, Stenlid R et al. Altered plasma levels of glucagon, GLP-1 and glicentin during OGTT in adolescents with obesity and type 2 diabetes. J Clin Endocrinol Metab 2016; 101: 1181–9.
  - ○ Early-life evidence that multiple proglucagon-derived peptides are dysregulated in obesity and T2DM, underscoring their role in disease development.
- English PJ, Ashcroft A, Patterson M, Dovey TM, Halford JC, Harrison J et al. Fasting plasma peptide-YY concentrations are elevated but do not rise postprandially in type 2 diabetes. Diabetologia 2006; 49: 2219–21.
  - ○ Identifies abnormal PYY dynamics in T2DM, with impaired postprandial rise despite elevated fasting levels, indicating a defect in satiety signaling.
- le Roux CW, Batterham RL, Aylwin SJ, Patterson M, Borg CM, Wynne KJ et al. Attenuated peptide YY release in obese subjects is associated with reduced satiety. Endocrinology 2006; 147: 3–8.
  - ○ Demonstrates diminished PYY secretion in obesity, linking gut hormone dysfunction directly with appetite dysregulation.
- Poykko SM, Kellokoski E, Horkko S, Kauma H, Kesaniemi YA, Ukkola O. Low plasma ghrelin is associated with insulin resistance, hypertension, and the prevalence of type 2 diabetes. Diabetes 2003; 52: 2546–53.
  - ○ One of the earliest studies connecting reduced ghrelin levels to insulin resistance and T2DM, establishing ghrelin as a metabolic biomarker.
- Ribeiro FM, Anderson M, Aguiar S, Gabriela E, Petriz B, Franco OL. Systematic review and meta-analysis of gut peptides expression during fasting and postprandial states in individuals with obesity. Nutr Res 2024; 127: 27 39.
  - ○ Recent synthesis showing broad alterations in gut peptide responses in obesity, highlighting variability across study designs and the need for standardized methodologies.
- Beglinger S, Meyer-Gerspach AC, Graf S, Zumsteg U, Drewe J, Beglinger C et al. Effect of a test meal on satiation hormones and their association with insulin resistance in obese adolescents. Obesity (Silver Spring) 2014; 22: 2047–52.
  - ○ Shows impaired meal-stimulated responses of satiety hormones in obese youth, linking gut peptide dynamics with early insulin resistance.
- Papamargaritis D, le Roux CW. Do gut hormones contribute to weight loss and glycaemic outcomes after bariatric surgery? Nutrients 2021; 13: 918.
  - ○ Reviews the evidence that changes in gut hormones, particularly GLP-1, PYY, and oxyntomodulin, mediate the metabolic and weight benefits of bariatric surgery.

## Data Availability

No datasets were generated or analysed during the current study.

## Abbreviations

AgRP/NPY: agouti-related peptide and neuropeptide Y
AMPK: AMP-activated protein kinase
AUCs: areas under the curve
BMI: body mass index
cAMP: cyclic AMP
CCK: cholecystokynin
CNS: central nervous system
DPP-4: dipeptidyl peptidase-4
GABA: gamma-aminobutyric acid
GCGR: G-protein-coupled receptor
GH: growth hormone
GHRH: growth hormone-releasing hormone
GHS-R: growth hormone secretagogue receptor
GIP: glucose-dependent insulinotropic polypeptide
GLP-1: glucagon-like peptide-1
GLP-1R: glucagon-like peptide-1 receptor
GLP-2: glucagon-like peptide-2
GLP-2R: glucagon-like peptide-2 receptor
GOAT: ghrelin O-acyltransferase
GRPP: glicentin-related pancreatic polypeptide
HOMA-IR: homeostatic model assessment of insulin resistance
HbA1C: glycated hemoglobin
IAPP: islet amyloid polypeptide
IGT: impaired glucose tolerance
IP-1: intervening peptide-1
iAUCs: incremental areas under the curve
MAFLD: metabolic dysfunction-associated fatty liver disease
MMT: mixed-meal test
MPGF: major proglucagon fragment
OGTT: oral glucose tolerance test
OXM: oxyntomodulin
PC: prohormone convertase
PGDPs: proglucagon-derived peptides
POMC: proopiomelanocortin
PP: pancreatic polypeptide
PYY: peptide YY
RAMPs: calcitonin receptor with receptor activity-modifying proteins
RIA: radioimmunoassay
RYGB: Roux-en-Y gastric bypass
SCT: secretin
SCTR: secretin receptor
SG: sleeve gastrectomy
T2DM: type 2 diabetes mellitus
